# Poem on Rheumatism

**Published:** 1855-05

**Authors:** E. S. S. Rouse


The following poem, which was written by a gifted poet and friend, we commend to the attention of our medical readers, although it first appeared in a secular paper. We are not made aware whether he had exhausted all the various therapeautic remedies before appealing to the *furies* as he is silent with regard to *colchicum*, *lemon acid*, *opium*, or *cotton-wool.* We are not too certain but that our talented friend is not somewhat addled with some of the *isms* in medicine, and when afflicted has neglected the most successful mode of relief. When that "*H—l o' a' diseases*," as he appears to regard it, assails him again, in his prayers let him place the above weapons in his magazine when he makes his onslaught.

Mr. Coggshall refers to it in the following language:

"The following poetical anathema, from our brother bard, Rouse, against the rheumatic pains—not the *room-attic* panes of poets, particularly, we suppose—must have been perpetrated under the most *inflammatory* provocation, for it is fiercer than a plaster of Spanish flies. Reader, remember that, if this is fun to you, it was frenzy to him."

### RHEUMATISM.

BY E. S. S. ROUSE.

Rheumatism! dreadful ism!
Fierce, tormenting, physical schism!
Demons take thee,
Furies rake thee,
Thunders shake thee,
Doctors break thee,
Plasters make thee
Quit thy ach-ee, ach-ee, ach-ee.

Do thou leave me!—don't decieve me,
Neither longer stay to grieve me,
If thou dost, then may thy ghost
In Tartarians' furnace roast;—
Harpies sieze thee,
Witchies tease thee,
Imps displease thee,
Pluto squeeze thee,
Boreas freeze thee,
Nothing ease thee,
Good forsake thee,
Ills o'ertake thee,
'Till they make thee
Quit thy ach-ee, ach-ee, ach-ee.

Unrelenting, cruel foe!
Do relieve me, let me go!
I detest thee,—do not stay!
Fly thou from me far away!
If thou art not dead to shame,
"Quit, O quit this mortal frame!"
Hie thee hence, to whence thou came,
Vengeful fiend of hateful name!
If thou wilt not go, I 'll drive thee;
In the devil's name I 'll hive thee,
Turn the key and bolt the door;
Rouse the fire and make thee roar;
Tell old Beelzebub to smoke thee,—
With the brimstone poker poke thee;

Genii daunt thee,
Goblins haunt thee,
Grim Cerberus rise and flaunt thee ;
Spirits smite thee,
Mad dogs bite thee,
Pleasures slight thee,
Doctors fight thee,
Evils smite thee,
Agues shake thee,
Horrors quake thee,
'Till they make thee
Quit thy ach-ee, ach-ee, ach-ee.

